# Efficient yeast cell-surface display of exo- and endo-cellulase using the *SED1* anchoring region and its original promoter

**DOI:** 10.1186/1754-6834-7-8

**Published:** 2014-01-14

**Authors:** Kentaro Inokuma, Tomohisa Hasunuma, Akihiko Kondo

**Affiliations:** 1 Department of Chemical Science and Engineering, Graduate School of Engineering, Kobe University, 1-1 Rokkodai, Nada, Kobe 657-8501, Japan; 2 Organization of Advanced Science and Technology, Kobe University, 1-1 Rokkodai, Nada, Kobe 657-8501, Japan; 3 Biomass Engineering Program, RIKEN, 1-7-22 Suehiro-cho, Tsurumi-ku, Yokohama, Kanagawa 230-0045, Japan; 4 Department of Food Bioscience and Technology, College of Life Sciences and Biotechnology, Korea University, Seoul 136-713, Republic of Korea

**Keywords:** *Saccharomyces cerevisiae*, Cell-surface display, β-glucosidase, Endoglucanase, Bioethanol production, Lignocellulosic biomass

## Abstract

**Background:**

The recombinant yeast strains displaying the heterologous cellulolytic enzymes on the cell surface using the glycosylphosphatidylinositol (GPI) anchoring system are considered promising biocatalysts for direct conversion of lignocellulosic materials to ethanol. However, the cellulolytic activities of the conventional cellulase-displaying yeast strains are insufficient for the hydrolysis of cellulose. In this study, we constructed novel gene cassettes for the efficient cellulose utilization by cellulase-displaying yeast strains.

**Results:**

The novel gene cassettes for the cell-surface display of *Aspergillus aculeatus* β-glucosidase (BGL1) and *Trichoderma reeseii* endoglucanase II (EGII) were constructed using the promoter and the GPI anchoring region derived from *Saccharomyces cerevisiae SED1*. The gene cassettes were integrated into the *S. cerevisiae* genome, then the β-glucosidase activity of these recombinant strains was evaluated. We revealed that simultaneous utilization of the *SED1* promoter and Sed1 anchoring domain in a gene cassette enabled highly-efficient enzyme integration into the cell wall. The β-glucosidase activity of recombinant yeast cells transduced with the novel gene cassette was 8.4-fold higher than that of a conventional strain. The novel EGII-displaying strain also achieved 106-fold higher hydrolysis activity against the water-insoluble cellulose than a conventional strain. Furthermore, direct ethanol production from hydrothermally processed rice straw was improved by the display of *T. reeseii* EGII using the novel gene cassette.

**Conclusions:**

We have developed novel gene cassettes for the efficient cell-surface display of exo- and endo-type cellulolytic enzymes. The results suggest that this gene cassette has the wide applicability for cell-surface display and that cellulase-displaying yeasts have significant potential for cost-effective bioethanol production from lignocellulosic biomass.

## Background

The search for practical petroleum substitutes from renewable resources has become a global priority as atmospheric carbon dioxide levels continue to rise. Lignocellulosic materials such as sugar cane bagasse, corn stover, rice straw, grasses, wood chips and other agricultural waste contain large amounts of polysaccharides such as cellulose and hemicellulose. These polysaccharides are one of the most abundant renewable resources in nature, and are attracting much attention as feedstocks for second-generation bioethanol production [1]. However, due to the recalcitrance of lignocellulosic materials, the pre-treatment requiring a large amount of energy and the large quantities of multiple enzymes are necessary for the complete hydrolysis of these polysaccharides to fermentable sugars [2-5]. The cost of the bioconversion of these materials to ethanol is much higher than that of conventional sugar sources such as corn starch and cane juice [1]. In particular, the cost of the enzymes is one of the biggest obstacles for commercially viable production of bioethanol from lignocellulosic materials. Therefore, consolidated bioprocessing (CBP), which combines enzyme production, saccharification of polysaccharides, and fermentation into a single process, has a great potential for the cost-effective production of ethanol from lignocellulosic materials [6-8]. To date, however, no natural microorganisms with the capability for efficient enzyme production, lignocellulose saccharification, and ethanol production have been identified.

Cellulose, a polymer of β*-*(1 to 4) linked glucose residues, is the major component of lignocellulosic materials (40 to 60% by total dry weight) [9]. *Saccharomyces cerevisiae* (*S. cerevisiae)* is the most commonly used microorganism for industrial ethanol production. Compared to bacteria, it has more rapid sugar consumption, provides a higher ethanol yield from glucose, and has higher resistance to ethanol and other compounds present in lignocellulosic hydrolysates [10,11]. However, native *S. cerevisiae* is not a candidate microorganism for the CBP of cellulose because it lacks the enzymes required for the hydrolyzation of cellulose into glucose. To address this problem, the heterologous expression and secretion of cellulolytic enzymes, including endoglucanase (EG), cellobiohydrolase (CBH) and β-glucosidase (BGL) from yeasts and other microorganisms, have been pursued over the last two decades [12]. On the other hand, efficient whole-cell biocatalysts for simultaneous saccharification and fermentation have also been constructed by displaying cellulolytic enzymes on the cell surface using the glycosylphosphatidylinositol (GPI) anchoring system [13-18]. The cell-surface engineering system has several industrial advantages over the enzyme secretion system. In this system, yeast is transformed by introducing fusion genes coding cellulolytic enzymes and the anchoring domain of GPI protein. The fused proteins self-immobilize into the yeast cell wall, so the activities of the enzymes are retained as long as the yeast continue to grow [19]. As the separation of the biocatalyst from the products is easy, reutilization of the yeast cells also enables re-use of the active enzymes displayed on the cell surface, without the need for the cells to reproduce. This approach would reduce the cost of yeast propagation as well as the cost of enzyme addition [20]. Furthermore, Matano *et al*. [21] recently reported a cell-surface display system that alleviated the adsorption of free cellulases onto the crystalline cellulose and improved the hydrolysis of cellulose and ethanol production.

In previous studies, the C-terminal domain of *α*-agglutinin (Sag1), which is a GPI protein in *S. cerevisiae*, has been mainly used as an anchoring domain in fusion proteins. The genes encoding these fusion proteins have been expressed by constitutive promoters such as *TDH3* and *PGK1* promoters. However, the cellulolytic activities on the cell surface provided using conventional gene cassettes are insufficient for the efficient CBP of cellulose. There is therefore, urgent need for improvements in yeast cell-surface display cassettes.

In this study, we constructed the novel gene cassettes for the cell-surface display of cellulolytic enzymes that incorporated the promoter and the GPI anchoring region derived from *S. cerevisiae SED1*, which encodes a major stress-induced structural GPI protein [22]. The gene cassettes for the cell-surface display of *Aspergillus aculeatus* β-glucosidase (BGL1) were integrated into the *HIS3* locus of the *S. cerevisiae* genome, then the BGL activity of these recombinant strains was evaluated. The results demonstrated that the simultaneous utilization of the *SED1* promoter and the anchoring region significantly improved BGL activity on the cell surface. In addition, we also constructed a recombinant yeast strain displaying the *Trichoderma reeseii* endoglucanase II (EGII) using this novel gene cassette. Hydrolysis activity against the water-insoluble cellulose of this strain also increased significantly. Furthermore, the ethanol productivity from hydrothermally processed rice straw was clearly improved by using this EGII-displaying strain.

## Results

### Construction of yeast strains

**Figure 1:**
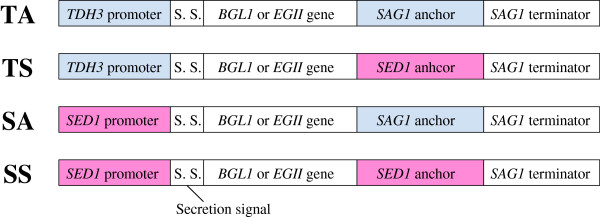
**Construction of novel gene cassettes for the yeast cell-surface display of cellulolytic enzymes.** All cassettes have the secretion signal sequence of the *Rhizopus. oryzae* glucoamylase gene and *SAG1* terminator.

A haploid yeast strain, *S. cerevisiae* BY4741 was used as the host strain in this study. Figure 1 shows the four types of gene cassettes constructed for cell-surface display of cellulolytic enzymes in this study. The TA cassette is a conventional cassette containing the *S. cerevisiae TDH3* promoter and the 3′-half of *S. cerevisiae SAG1* as a GPI anchoring region. The novel gene cassettes are hereinafter referred to as TS (*TDH3* promoter and *S. cerevisiae SED1* anchoring region), SA (*SED1* promoter and *SAG1* anchoring region), and SS cassettes (*SED1* promoter and *SED1* anchoring region). All cassettes have the secretion signal sequence of the *Rhizopus oryzae* glucoamylase gene and *SAG1* terminator. The plasmids with these cassettes (Table 1) were integrated into the *HIS3* locus of the chromosomal DNA by homologous recombination. The constructed strains listed in Table 2 were used for the experiments described below.

**Table 1:** Characteristics of the integrative plasmids used in this study

**Plasmids**	**Relevant genotype**	**Source/references**
pRS403	*HIS3* Integrative vector without display cassette	Agilent Technologies
pδU-PGAGEG	*URA3* δ-Integrative vector with cell-surface display cassette of *T. reesei EGII*	[26]
pIBG13	*HIS3* Integrative vector of cell-surface display cassette with *TDH3* promoter, *SAG1* anchoring region and *SAG1* terminator	[43]
pIBG13S	*HIS3* Integrative vector of cell-surface display cassette with *TDH3* promoter, *SED1* anchoring region and *SAG1* terminator	This study
pISBG13	*HIS3* Integrative vector of cell-surface display cassette with *SED1* promoter, *SAG1* anchoring region and *SAG1* terminator	This study
pIBG-TA	*HIS3 TDH3*_*P*_*-A. aculeatus BGL1-SAG1*_*A*_*-SAG1*_*T*_	This study
pIBG-TS	*HIS3 TDH3*_*P*_*-A. aculeatus BGL1-SED1*_*A*_*-SAG1*_*T*_	This study
pIBG-SA	*HIS3 SED1*_*P*_*-A. aculeatus BGL1-SAG1*_*A*_*-SAG1*_*T*_	This study
pIBG-SS	*HIS3 SED1*_*P*_*-A. aculeatus BGL1-SED1*_*A*_*-SAG1*_*T*_	This study
pIEG-TA	*HIS3 TDH3*_*P*_*-T. reesei EGII -SAG1*_*A*_*-SAG1*_*T*_	This study
pIEG-TS	*HIS3 TDH3*_*P*_*-T. reesei EGII -SED1*_*A*_*-SAG1*_*T*_	This study
pIEG-SA	*HIS3 SED1*_*P*_*-T. reesei EGII –-SAG1*_*A*_*-SAG1*_*T*_	This study
pIEG-SS	*HIS3 SED1*_*P*_*-T. reesei EGII –SED1*_*A*_*-SAG1*_*T*_	This study

T. reesei, Trichoderma reesei; A. aculeatus, Aspergillus aculeatus.

**Table 2:** Characteristics of the yeast strains used in this study

**Strains**	**Relevant genotype**	**Source**
BY4741	*MATa his3Δ1 leu2Δ0 met15Δ0 ura3Δ0*	Life Technologies
BY-403	BY4741/pRS403	This study
BY-BG-TA	BY4741/pIBG-TA	This study
BY-BG-TS	BY4741/pIBG-TS	This study
BY-BG-SA	BY4741/pIBG-SA	This study
BY-BG-SS	BY4741/pIBG-SS	This study
BY-EG-TA	BY4741/pIEG-TA	This study
BY-EG-TS	BY4741/pIEG-TS	This study
BY-EG-SA	BY4741/pIEG-SA	This study
BY-EG-SS	BY4741/pIEG-SS	This study

### Transcriptional levels of *BGL1* and BGL activities of recombinant yeast strains

**Figure 2:**
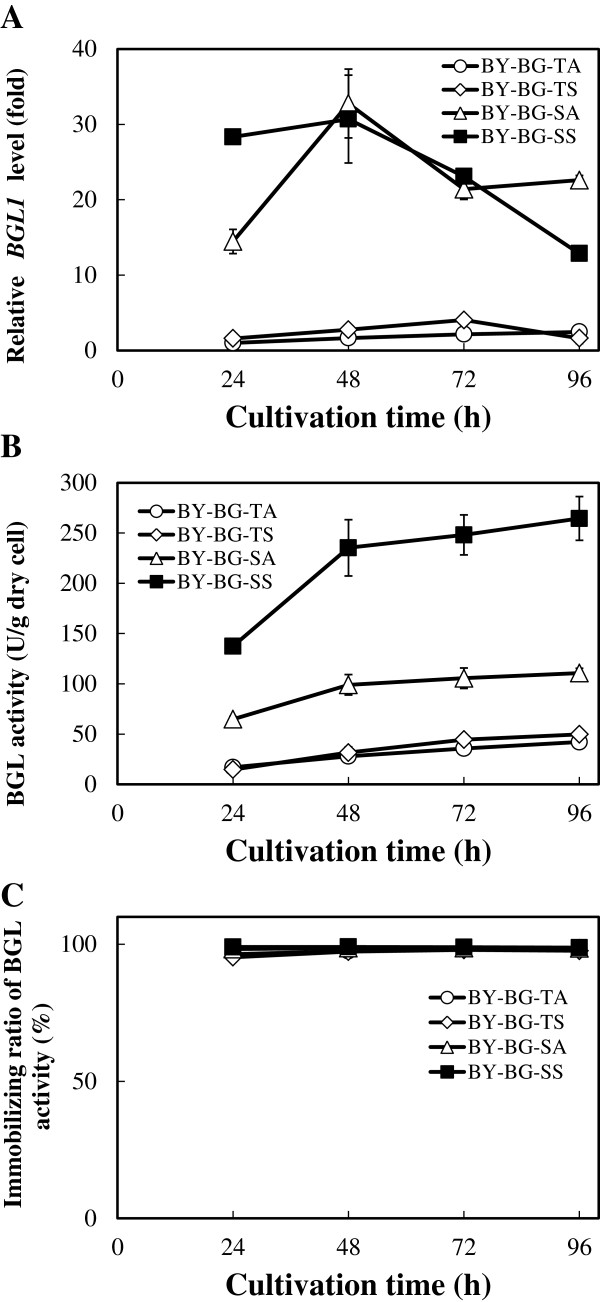
**Characterization of β-glucosidase (BGL)-displaying yeast strains. (A)** Time course of transcriptional levels of *BGL1*. The relative *BGL1* level of each sample is shown as a fold change in the mRNA level from the average of BY-BG-TA in 24 h. **(B)** Time course of BGL activities. **(C)** Immobilizing ratios of BGL activity. The immobilizing ratio was calculated by dividing the activity of the cell by the total activity of the culture medium, including the culture supernatant. Error bars indicate the standard deviations of three independent experiments.

β-glucosidase (BGL) cleaves cello-oligosaccharides from the non-reducing end to give glucose in the last step of enzymatic cellulose degradation. This enzyme is required for the complete hydrolysis of cellulose. Recombinant strains harboring gene cassettes for cell-surface display of *A. aculeatus* BGL1 (BY-BG-TA, TS, SA and SS strains in Table 2) were cultivated aerobically for 96 h. The culture broth was sampled every 24 h, and the transcriptional level of *BGL1* and BGL activity in the cell was investigated as described in Methods. Throughout the cultivation period, the transcriptional levels of *BGL1* controlled by the *SED1* promoter (BY-BG-SA and SS) were far higher than that of the *TDH3* promoter (BY-BG-TA and TS) (Figure 2A). Whereas *BGL1* expression controlled by the *TDH3* promoter gradually increased during 72 h of cultivation, the expression under the control of the *SED1* promoter rose dramatically and rapidly during the first 48 h of cultivation.

BGL activity of BY-BG-TA (28 ± 3 U/g dry cells at 48 h) was slightly improved by the replacement of the GPI anchoring domain by the Sed1 protein (BY-BG-TS, 32 ± 6 U/g dry cell at 48 h). Promoter replacement by the *SED1* promoter resulted in significantly improved BGL activity (BY-BG-SA). BGL activity of BY-SBGA reached 99 ± 10 U/g dry cells after 48 h cultivation (Figure 2B). Furthermore, simultaneous utilization of the *SED1* promoter and Sed1 anchoring domain (BY-BG-SS) achieved more than double the BGL activity of BY-BG-SA (235 ± 28 U/g dry cells at 48 h) to approximately 8.4-fold higher than that of the conventional strain, BY-BG-TA. These results suggested a synergetic effect of the *SED1* promoter and Sed1 anchoring domain for the cell-surface display of enzymes. In all strains investigated, most of the BGL activity was immobilized on the yeast cell surface and only a small amount of activity was detected in the culture supernatants throughout the cultivation (Figure 2C). No significant difference was observed in the cell growth of the various strains (data not shown).

### Kinetic parameters of BGL displayed on the cell surface

**Figure 3:**
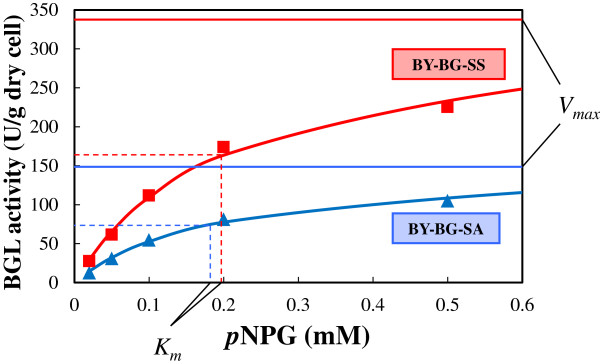
**Kinetic parameters of the β-glucosidase (BGL)-displaying strains.** After 48 h cultivation, BGL activity of BY-BG-SA and SS cells was measured between 0.02 and 0.5 mM *p*-nitrophenyl-β-D-glucopyranoside (*p*NPG) at pH 5.0 and 30°C. *K*_*m*_ and *V*_max_ values were calculated by weighted non-linear least-squares analysis of the raw data [23]. SA, *SED1* promoter and *SAG1* anchoring region; SS, *SED1* promoter and *SED1* anchoring region.

To characterize the BGL activities of BY-BG-SA and SS, their kinetic parameters were determined. For the determination of the *K*_*m*_ and *V*_max_, BGL activity was measured between 0.02 and 0.5 mM *p*-nitrophenyl-β-D-glucopyranoside (*p*NPG) (Figure 3). Kinetic parameters were calculated by weighted non-linear least-squares analysis of the raw data to fit to the Michaelis-Menten function [23]. The *V*_max_ values of BGL activity for *p*NPG cleavage in BY-BG-SA and SS strains were 148 and 326 U/g dry cells, respectively (Figure 3). In contrast, no significant difference was observed in the *K*_*m*_ value of these strains (0.181 and 0.198 mM, respectively). Generally in enzyme kinetics, such a change in kinetic parameters is due to changes in the amount of active enzyme per unit of protein weight. As the measurement unit of BGL activity is defined as per unit cell-weight (U/g dry cells) in this study, these results suggest that the amount of active enzyme per unit cell-weight differs substantially between BY-BG-SA and SS, and it accounts for the difference in BGL activity.

### Cell-surface display of *T. reeseii* EGII

Multiple enzymes, including endo-type cellulases, are necessary for the hydrolysis of cellulose [5]. EG, which cleaves glycosidic linkages randomly along cellulose chains, is also one of the enzymes required for the complete hydrolysis of cellulose, as is BGL. To test the applicability of the *SED1* promoter and the anchoring region for cell-surface display of an endo-type cellulase, *T. reesei* EGII-displaying yeast strains BY-EG-TA, TS, SA and SS were constructed (Table 2). These strains were cultured under aerobic conditions at 30°C for 48 h, then the enzyme activity of the cells against water-insoluble substrates was evaluated using azurine cross-linked hydroxyethylcellulose (AZCL-HE-Cellulose). The results are shown in Table 3. The *SED1* promoter had a positive effect on the hydrolysis of AZCL-HE-Cellulose, as it had for the BGL activity (Figure 2B). The AZCL-HE-Cellulose hydrolysis activity of BY-EG-TA was significantly improved by using the Sed1 anchoring domain (BY-EG-TS), although there was only a small increase in BGL activity between BY-BG-TA and TS (Figure 2B). The hydrolysis activity of BY-EG-TS was 72-fold higher than that of the conventional strain BY-EG-TA. Furthermore, BY-EG-SS showed 106-fold higher activity against AZCL-HE-Cellulose than BY-EG-TA. Although a measurement of AZCL-HE-Cellulose hydrolysis activity does not provide absolute values of EG activity [24], these results indicated that the gene cassettes carrying the Sed1 anchoring domain are more favorable for the cell-surface display of endo-type cellulases than those with the Sag1 anchoring domain.

**Table 3:** AZCL-HE-Cellulose hydrolysis activity of EGII-displaying yeast strains

**Strains**	**EG activities (A**_**590**_**)**^**a**^
BY-EG-TA	0.030 ± 0.004
BY-EG-TS	2.157 ± 0.361
BY-EG-SA	0.053 ± 0.015
BY-EG-SS	3.188 ± 0.133

^a^Enzyme activity was evaluated based on the absorbance at wavelength of 590 nm in a 1 cm optical path length cuvette (A_590_) of the blue dye released into the supernatant after incubation for 4 h at 38°C. The averages for three independent experiments are shown with their standard deviations.

### Direct ethanol production from hydrothermally processed rice straw

**Figure 4:**
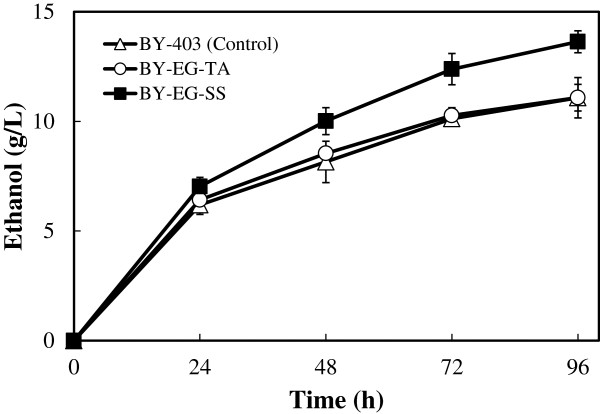
**Time course of direct ethanol production from 100 g dry weight/L of hydrothermally processed rice straw.** Error bars indicate the standard deviations of three independent experiments.

In order to evaluate the effects of the cell-surface display of EGII on the hydrolysis of lignocellulosic biomass, direct ethanol production from hydrothermally processed rice straw was performed using the cells of EGII-displaying strains BY-EG-TA and SS. One filter paper unit (FPU)/g-biomass of commercial cellulase cocktail (Cellic CTec2) was added to the fermentation mixture to supply other cellulolytic enzymes. Fermentation using the BY-403 strain, which was transformed with an empty vector (pRS403), was also carried out as a control. As shown in Figure 4, no significant difference was observed in ethanol productivity from the pretreated rice straw between BY-EG-TA and the control strain (11.1 ± 0.6 and 11.1 ± 0.9 g/L of ethanol after 96 h, respectively). On the other hand, the ethanol productivity of this fermentation was clearly improved by using BY-EG-SS strain (13.6 ± 0.5 g/L of ethanol after 96 h).

## Discussion

In this study, we developed novel gene cassettes for the efficient cell-surface display of cellulolytic enzymes using the *S. cerevisiae SED1* promoter and anchoring region (Figure 1). BGL-displaying strains using the novel gene cassettes (BY-BG-TS, SA and SS) achieved higher BGL activity on the cell surface than a conventional strain (BY-BG-TA) harboring a cassette carrying the *TDH3* promoter and the *SAG1* anchoring region (Figure 2B). The BY-BG-SS strain, which had a gene cassette with the *S. cerevisiae SED1* promoter and its anchoring region (SS cassette), achieved the highest BGL activity (Figure 2B). Comparison of GPI anchoring domains for effective cell-surface display using the inducible *GAL7* promoter has been reported [25]. However, there have been no reports on the effect of the combinations of a promoter and an anchoring domain for enzyme cell-surface display. To our knowledge, this is the first report on the synergetic effect of a GPI anchoring domain and its original promoter for the cell-surface display.

Previously, the activity of heterologous enzymes displayed on a yeast cell surface was improved by increasing the copy number of the gene cassettes, for example by using the multi-copy genome integration method (δ-integration) [26]. However, the enzyme activity of recombinant strains constructed by the δ-integration method is relatively unstable, due to the random genome integration of gene cassettes. Yamada *et al*. [26] constructed a recombinant *S. cerevisiae* strain, MT8-1/δBEC, by δ-integration. MT8-1/δBEC had six copies of a gene cassette for BGL display using the *PGK1* promoter and the Sag1 anchoring domain. MT8-1/δBEC achieved a BGL activity of 15 U/g wet cells, which is comparable to approximately 100 U/g dry cells after 72 h cultivation. On the other hand, the BGL activity of BY-BG-SS reached 235 ± 28 U/g dry cells after only 48 h cultivation, even though it carried only a single gene cassette for BGL display. Although these strains cannot be compared directly, because MT8-1 and BY4741 strains have different genetic backgrounds, this novel gene cassette will thus allow the more stable construction of a heterologous enzyme-displaying yeast strain with high activity. The BGL activity of BY-BG-SS (235 ± 28 U/g dry cells after 48 h) is close to the specific activity of purified *A. aculeatus* BGL1 (280.7 U/g protein) at pH 5.0 and 30°C [27]. The *K*_*m*_ value of the BGL on the BY-BG-SS strain for *p*NPG (0.198 mM) is lower than that of purified *A. aculeatus* BGL1 (0.23 mM) [27]. Furthermore, SS cassettes also have a significant effect on the cell-surface display of *T. reeseii* EGII, which is an endo-type cellulolytic enzyme. Hydrolysis activity of BY-EG-SS against the water-insoluble substrate, AZCL-HE-Cellulose, was 106-fold higher than that of the conventional strain, BY-EG-TA (Table 3). These results suggest that recombinant yeast cells displaying cellulolytic enzymes using SS cassettes are highly promising biocatalysts for efficient ethanol production from cellulosic materials.

It has been reported that the *SED1* expression level increases during the stationary phase [22]. As shown in Figure 2A, the expression level of the BGL1 controlled by *SED1* promoter rose dramatically and rapidly after 48 h cultivation. The BGL1 activity of BY-BG-SA and SS was 3.5- and 7.4-fold higher than that of BY-BG-TA and TS after 48 h, respectively (Figure 2B). The high expression level of *BGL1* controlled by the *SED1* promoter is likely one of the causes for the high BGL activity of the BY-BG-SA and SS strains. On the other hand, it was previously reported that the mRNA level of *SED1* varies periodically during the cell cycle and that the peak of *SED1* expression is M phase, whereas no apparent peak was observed in *TDH3* expression during the cell cycle [28]. In the cell cycle of *S. cerevisiae*, the synthesis of the new cell wall is activated in the M phase. The expression of many genes involved in the synthesis of cell-wall components and their delivery also increases in this phase [28], suggesting that the M phase is the most suitable for efficient cell wall integration of GPI proteins. Although further investigation using other constitutive promoters such as *PGK1* promoter is needed, the cell cycle-regulated expression controlled by the *SED1* promoter might be also one of the causes of high BGL activity of the BY-BG-SA and SS strains (Figure 2B).

The result shown in Figure 3 suggests that the amount of BGL1 immobilized in the cell wall varies depending on the C-terminal anchoring domain, and accounts for the difference in BGL activity between the BY-BG-SA and SS strains. It has been estimated that *S. cerevisiae* cell contains the 60 GPI proteins, and most of them can be integrated into the cell wall by a covalent attachment to the β-(1 to 6) glucan chains in the glucan layer [29]. In recombinant yeasts, heterologous GPI-anchoring fusion proteins must compete with the native GPI proteins for the limited protein incorporation capacity of the glucan layer [25]. Therefore, it is possible that the integration efficiency of GPI-anchoring enzymes strongly affects the population and activity of the enzymes in the cell wall [25]. It was recently reported that different GPI proteins exhibit different transportation and integration efficiencies into the cell wall [30-34]. Analysis of a large panel of biochemically verified GPI proteins suggested that specific residues in the upstream region of the ω-site, which is the cleavage/attachment site of the C-terminal GPI-anchoring signal recognized by a GPI transamidase, seem to favor cell wall localization [32-34]. Hamada *et al*. [33] reported that the amino acid sequence in the upstream region of ω-site differed substantially between Sed1 and Sag1, and that reporter protein fused with the Sed1 anchoring domain had higher cell-wall integration efficiency than protein fused with the Sag1 anchoring domain. The results shown in Figures 2 and 3 are in good agreement with these reports.

Another possible cause for the high BGL activity of the BY-BG-SS strain is the specific synergy of the *SED1* promoter with the Sed1 anchoring domain in SS cassette. We constructed novel gene cassettes for BGL1 display with the *S. cerevisiae CWP2* promoter and/or its anchoring region based on the SS cassette (Additional file 1). Cwp2 is a major GPI protein in *S. cerevisiae,* as is Sed1 [35]. The mRNA peak level of *CWP2* during the cell cycle occurs during the G2 phase [28]. BGL-displaying strains harboring these cassettes (BY-BG-SC, CS and CC) were constructed and their BGL activity was evaluated (Additional file 2). BY-BG-CC achieved the highest BGL activity among these strains (148 ± 1 U/g dry cells after 48 h), whereas BY-BG-SC exhibited less than half the BGL activity of BY-BG-CC (71 ± 2 U/g dry cells after 48 h) (Additional file 2). These results suggest that each gene encoding GPI anchoring protein is expressed most efficiently at different phases during the cell cycle, as regulated by its original promoter in *S. cerevisiae* cells. After the attachment of the GPI anchor in the endoplasmic reticulum (ER), GPI proteins leave the ER in COPII-coated vesicles and travel via the Golgi to the plasma membrane [36]. During this intracellular transportation, each GPI protein and its GPI anchors are subject to a variety of modifications [29]. Although many aspects of the intracellular transportation and modification systems for each GPI protein remain unclear [29], cell cycle-regulated expression of a gene encoding GPI protein under the control of its original promoter might favor efficient intracellular transportation and modification. Further validation for other GPI proteins and their promoters is needed to prove this synergetic effect. Regardless, the simultaneous utilization of an anchoring domain of GPI protein and its original promoter is a good option for constructing gene cassettes providing efficient cell-surface display.

The cell wall of yeast is made of a thick microfibrillar array of β-(1 to 3) glucan and β-(1 to 6) glucan chains [37]. It is possible that a part of GPI anchoring enzymes expose their catalytic domain to external surface of the cell wall while the remainder is completely embedded in the glucan layer [25]. Small substrates such as cellobiose are accessible to all integrated enzymes because these substrates penetrate the cell wall. In contrast, large substrates such as cellulose can only access enzymes exposed on the external surface. Therefore, the hydrolysis of highly polymerized substrates by enzymes integrated into the yeast cell wall is more difficult than hydrolysis of small substrates [25,38]. In this study, we constructed several *T. reeseii* EGII-displaying strains (BY-EG-TA, TS, SA and SS) and evaluated the enzyme activity of these cells using AZCL-HE-Cellulose as the water-insoluble substrate. Whereas BY-EG-TA and SA had very little activity against AZCL-HE-Cellulose, BY-EG-TS and SS showed significant activity (Table 3). This result suggests that the Sed1 anchoring domain provides significant advantages over the Sag1 anchoring domain not only in the efficiency of cell wall integration, but also in localizing the enzyme at the external surface of the cell wall. We constructed the novel gene cassette for EGII display with the *S. cerevisiae CWP2* promoter and its anchoring region (Additional file 1). The EG-displaying strain harboring this cassette (BY-EG-CC) was constructed and the hydrolysis activity against AZCL-HE-Cellulose was evaluated. The hydrolysis activity of BY-EG-CC (A_590_ = 0.021 ± 0.001) was lower than BY-EG-TA (0.030 ± 0.004), even though BGL-displaying strain using the CC cassette (BY-BG-CC) achieved high BGL activity (Additional file 2). This result also suggests that the anchoring domain has significant effect on the localization of enzymes in the cell wall. On the other hand, the possibility of the effect of the steric hindrance by different anchoring domains fused with EGII cannot be excluded. Although the full length of Sed1 was used as the Sed1 anchoring domain in this study, it has been reported that C-terminal-229 amino acids of Sed1 can be used as an anchoring domain [25]. It cannot be denied that the extra N-terminal sequence of Sed1 could function as a linker, causing the positive effect of the substrate accessibility of EGII. Investigation of the surface accessibility of the displayed enzymes by other experiments such as immunofluorescence labeling [39] would be necessary for more understanding about the localization of Sed1 in the cell wall.

*SED1* is a major stress-induced gene in *S. cerevisiae,* and the expression level increases during stressful industrial fermentation such as sake brewing [22,40]. Hasunuma and Kondo [1] suggested that the environment of an economically feasible CBP using cellulase-displaying yeast strains must also be stressful (highly concentrated yeast cells, high osmotic pressure, high temperature, low nutrition capacity and fluctuating processes). Therefore, the stress-induced property of the *SED1* gene should be favorable for the cell-surface display of cellulolytic enzymes in CBP. On the other hand, the transcriptional levels of *BGL1* controlled by the *SED1* promoter decreased dramatically after 48 h cultivation (Figure 2A). This result suggested that any factors other than stress regulate the expression of *SED1* promoter. Identification of the regulatory factors in the expression of *SED1* promoter would be necessary for further enhancement of the cellulolytic activities on the cell surface provided using the SS cassette.

In the direct ethanol production from hydrothermally processed rice straw, the BY-EG-SS strain enhanced the saccharification and fermentation of the pretreated rice straw in the presence of a commercial cellulase cocktail (Figure 4). EGII was displayed in the cell surface of the BY-EG-SS strain and successfully compensated for cellulolytic activity of the commercial enzymes under stressful conditions. As stated in the Background Section, the cost of commercial enzymes is one of the biggest obstacles to cost-effective bioethanol production from lignocellulosic materials. The result shown in Figure 4 indicates that cellulase-displaying yeast strains using the SS cassette have significant potential for reducing the cost of commercial enzymes and for the cost-effective CBP of lignocellulosic biomass.

It has been reported that the synergetic effects of EG, CBH and BGL are necessary for the effective hydrolyzation of cellulose using cell-surface-displaying yeasts, and that optimization of the ratios of the activities of these enzymes is also important for the hydrolyzation efficiency [26,41,42]. The BY-EG-SS strain clearly improved the ethanol productivity from pretreated rice straw, but this strain expressed only *EGII* (Figure 4). The cellulolytic activity of this strain could increase with the additional display of other enzymes in the future. In addition, we revealed that the combination of the promoter and anchoring domains in the gene cassette has significant effects on the efficiency of enzyme display. If the activity of various enzymes on the cell surface can be controlled artificially by the combinations of their promoters and anchoring domains, we will be able to construct the recombinant yeast strains optimized for the CBP of lignocellulosic materials.

## Conclusions

We have developed novel gene cassettes for the efficient cell-surface display of exo- and endo-type cellulolytic enzymes. We demonstrated that the combinations of a promoter and an anchoring domain in the display cassette had significant effects on the efficiency with which the enzyme integrated into the cell wall. Simultaneous utilization of the *SED1* promoter and Sed1 anchoring domain in a gene cassette enabled highly efficient enzyme integration into the cell wall. The BGL-displaying yeast strain carrying this cassette achieved high BGL activity within a relatively short cultivation time. Furthermore, this novel display cassette is also suitable for the hydrolyzation of insoluble substrates including lignocellulosic biomass. Ethanol productivity from hydrothermally processed rice straw was clearly improved by using the novel gene cassettes for EGII display. If cell-surface cellulolytic enzyme activities can be controlled and optimized artificially by the combinations of the promoter and anchoring domain, then cellulase-displaying yeasts will be an attractive option for cost-effective CBP of lignocellulosic materials.

## Methods

### Strains and media

The host for recombinant DNA manipulation was the *Escherichia coli* (*E. coli*) strain NovaBlue (Novagen, Madison, WI, USA). *E. coli* transformants were grown in Luria-Bertani medium (10 g/L of tryptone, 5 g/L of yeast extract and 5 g/L of sodium chloride) supplemented with 100 μg/ml of ampicillin at 37°C. The genetic properties of all yeast strains used in this study are summarized in Table 2. Cell-surface display cassettes of cellulase were expressed in the haploid yeast strain *S. cerevisiae* BY4741 (LifeTechnologies, Carlsbad, CA, USA). The yeast transformants were screened and pre-cultured in synthetic dextrose (SD) medium (6.7 g/L of yeast nitrogen base without amino acids (Difco Laboratories, Detroit, MI, USA) and 20 g/L of glucose) supplemented with appropriate amino acids and nucleic acids in a shaker incubator (180 rpm) at 30°C, and then aerobically cultured in yeast extract peptone dextrose (YPD) medium (10 g/L of yeast extract, 20 g/L of Bacto-peptone (Difco Laboratories) and 20 g/L of glucose) in a shaker incubator (150 rpm) at 30°C. Yeast cells were harvested by centrifugation at 1,000 × g for 5 minutes, then washed twice with distilled water followed by centrifugation at 1,000 × g for 5 minutes. Wet cell-weight of the washed yeast cells was determined by weighing the cell pellet. The estimated dry cell-weight for all strains was approximately 0.15-fold the wet cell-weight. The cell pellets were used for the enzyme assays and quantification of transcriptional level as described below.

### Plasmid construction and yeast transformation

The plasmids and primers used in this study are summarized in Tables 1 and 4, respectively. The 3′ half of *SAG1* cording region (963 bp) and the full length of *SED1* cording region except for the start codon (1,014 bp) were used as *SAG1* and *SED1* anchoring regions, respectively. The integrative plasmids for cell-surface display with *TDH3* promoter were constructed as follows: the DNA fragment encoding *A. aculeatus* BGL1 was amplified from plasmid pIBG13 [43] by PCR using the BGL1-F and BGL1-PG-R primers. Similarly, the DNA fragments encoding the anchoring domains of *S. cerevisiae* Sag1 and Sed1 were amplified from *S. cerevisiae* BY4741 [44] genomic DNA by PCR using the SAG1a-PG-F and SAG1a-R primers and SED1a-PG-F and SED1a-BsrGI-R primers, respectively. These anchor fragments were connected to the 3′-terminus of the *BGL1* fragment by the fusion PCR method [45] using the BGL1-F and SAG1a-R primers and BGL1-F and SED1a-BsrGI-R primers, respectively. These fragments encoding BGL1-Sag1 and BGL1-Sed1 were digested with *Nco*I and *Bsr*GI, and then subcloned into the *Nco*I and *Bsr*GI sites of pIBG13. The resulting plasmids were named pIBG-TA and pIBG-TS, respectively. The *Xho*I*-Bsr*GI DNA fragment encoding the Sed1 anchoring domain was amplified from *S. cerevisiae* BY4741 genomic DNA by PCR using the SED1a-XhoI-F and SED1a-BsrGI-R primers. This fragment was subcloned into the *Xho*I and *Bsr*GI sites of pIBG13. The resulting plasmid was named pIBG13S. The *Nco*I*-Xho*I DNA fragment encoding *T. reeseii* EGII was amplified from pδU-PGAGEG [26] by PCR using the EGII-NcoI-F and EGII-XhoI-R primers. This fragment was subcloned into the *Nco*I and *Xho*I sites of pIBG13 and pIBG13S. The resulting plasmids were named pIEG-TA and pIEG-TS, respectively.

**Table 4:** PCR primers used in this study

**Primers**	**Sequence**
BGL1-F	atgcaactgttcaatttgcc
BGL1-PG-R	gggcccgggcccgggttgcaccttcgggagc
SAG1a-PG-F	cccgggcccgggcccagcgccaaaagctctt
SAG1a-R	taaaatctgcggtgagacgg
SED1a-PG-F	cccgggcccgggcccaaattatcaactgtcctattatctgc
SED1a-BsrGI-R	gccatctgtacattataagaataacatagcaacaccag
SED1a-XhoI-F	gccatcctcgagtaaattatcaactgtcctattatctgc
EGII-NcoI-F	gccatcccatgggtcagcagactgtctggggc
EGII-XhoI-R	gccatcctcgagccctttcttgcgagacacgag
SED1p-CBA-F	cctcttcgctattacgccagattggatatagaaaattaacgtaaggc
SED1p-CBA-R	ggcaaattgaacagttgcatcttaatagagcgaacgtatttt
VS-CBA-F	aatacgttcgctctattaagatgcaactgttcaatttgcc
VS-CBA-R	gttaattttctatatccaatctggcgtaatagcgaagagg

The integrative plasmids with the *SED1* promoter were constructed as follows: The DNA fragment encoding the *SED1* promoter region was amplified from *S. cerevisiae* BY4741 genomic DNA by PCR using the SED1p-CBA-F and SED1p-CBA-R primers. Likewise, the vector fragment was amplified from pIBG13 by PCR using the VS-CBA-F and VS-CBA-R primers. These fragments were connected using the isothermal assembly method [46]. The resulting plasmid was named pISBG13. The *Nco*I*-Bsr*GI DNA fragments encoding BGL1-Sag1, BGL1-Sed1, EGII-Sag1 and EGII-Sed1 were obtained from plasmids pIBG-TA, pIBG-TS, pIEG-TA and pIEG-TS, respectively. Then, these fragments were subcloned into the *Nco*I and *Bsr*GI site of pISBG13. The resulting plasmids were named pIBG-SA, pIBG-SS, pIEG-SA and pIEG-SS, respectively.

Each plasmid was digested with *Nde*I within the *HIS3* gene. The linearized plasmids were then transformed into *S. cerevisiae* BY4741 using the lithium acetate method [47], and integrated into the *HIS3* locus of the chromosomal DNA by homologous recombination, respectively.

### Enzyme assays

Enzyme activities of the yeast cells were evaluated as described below. BGL activity was assayed with *p*NPG as the substrate. Washed yeast cells were resuspended in water (1 g wet cells/L): 100 μL of the suspended cell or culture supernatant was added to 400 μL of 2 mM *p*NPG solution dissolved in 50 mM sodium citrate buffer, pH 5.0. After incubation for 10 minutes in a shaker incubator (500 rpm) at 30°C, 500 μL of 3.0 M sodium carbonate solution was added to terminate the reaction, and the *p*-nitrophenol liberated was determined based on the absorbance at 400 nm. For these conditions, the molar extinction coefficient of 18,720 M^-1^ cm^-1^ was used to calculate the concentration of *p*-nitrophenol in the assay mixture. One unit of enzyme activity was defined as the amount of enzymes required to liberate 1 μmol of *p*-nitrophenol per minute.

EG activity was assayed with AZCL-HE-Cellulose, which is a purified insoluble polysaccharide as the substrate. The substrate is supplied commercially in a tablet form as Cellazyme C tablets (Megazyme, Bray, Ireland). Washed yeast cells were resuspended in water (50 g wet cells/L), then 500 μL of the cell suspension was added to 2 mL of assay solution containing a Cellazyme C tablet and 50 mM sodium citrate buffer, pH 5.0. After incubation for 4 h at 38°C, endo-β-1,4-glucanase activity was evaluated based on the absorbance at 590 nm of the blue dye released into the supernatant.

### Quantification of the transcriptional level of the *BGL1* gene by real-time PCR

The transcriptional level was quantified by real-time quantitative-PCR as described previously [48]. The PCR primers BGL 761F and BGL 858R [26] were used for quantification of transcriptional level of *BGL1. ACT1* was used as the internal standard.

### Direct ethanol production from hydrothermally processed rice straw

Hydrothermally processed rice straw purchased from Mitsubishi Heavy Industries, Ltd. (Tokyo, Japan) was used as a cellulosic material. Ethanol fermentation of the cellulosic material was performed in a 50 mL polypropylene tube (Corning Inc., Corning, NY, USA) and a heat block (Thermo Block Rotator SN-06BN; Nissin, Tokyo, Japan) as described previously [49]. *S. cerevisiae* strains used for fermentation were grown under aerobic conditions at 30°C for 48 h in 500 mL YPD medium. The yeast cells were collected by centrifugation at 1000 × g for 10 minutes at 20°C, then washed twice with distilled water. The cells were then resuspended in 10 mL yeast extract peptone (YP) medium (10 g/L of yeast extract and 20 g/L of Bacto-peptone) containing 50 mM sodium citrate buffer (pH 5.0), 100 g/L of cellulosic material and 1 FPU/g-biomass of commercial cellulase (Cellic CTec2; Novozymes Inc., Bagsvaerd, Denmark) at an initial cell concentration of 100 g wet cells/L. Fermentation was initiated by the addition of yeast cells into the tube followed by axial rotation at 35 rpm at 38°C. The ethanol concentration in the fermentation medium was determined by HPLC (Shimadzu, Kyoto, Japan), as described previously [50].

## Abbreviations

AZCL-HE-Cellulose: azurine cross-linked hydroxyethylcellulose; BGL: β-glucosidase; bp: base pairs; CBH: cellobiohydrolase; CBP: consolidated bioprocessing; EG: endoglucanase; ER: endoplasmic reticulum; FPU: filter paper unit; GPI: glycosylphosphatidylinositol; pNPG: *p*-nitrophenyl-β-D-glucopyranoside; SA: *SED1* promoter and *SAG1* anchoring region; SS: *SED1* promoter and *SED1* anchoring region; TA: *TDH3* promoter and *SAG1* anchoring region; TS: *TDH3* promoter and *SED1* anchoring region.

## Competing interests

The authors declare that they have no competing interests.

## Authors’ contributions

KI designed and performed the experiments, and wrote the manuscript. TH helped to draft the manuscript. TH and AK supervised the research. All authors read and approved the final manuscript.

## Supplementary Material

Additional file 1: Figure S1 Construction of novel gene cassettes with the *S. cerevisiae CWP2* promoter and/or its anchoring region. The novel gene cassettes with the *S. cerevisiae CWP2* promoter and/or its anchoring region were constructed based on the SS cassette for the yeast cell-surface display of β-glucosidase (BGL1). All cassettes have the secretion signal sequence of the *R. oryzae* glucoamylase gene and *SAG1* terminator. Click here for file

Additional file 2: Figure S2 Time course of β-glucosidase (BGL) activities of BGL-displaying strains (BY-BG-SC, CS and CC). Recombinant strains harboring gene cassettes shown in Additional file 1: Figure S1 were cultured under aerobic conditions at 30°C for 96 h. The culture broth was sampled every 24 h, and BGL activity in the cell was investigated as described in the Methods. Error bars indicate the standard deviations of three independent experiments. Click here for file
