# Supplementary figures and images for: Efficient yeast cell-surface display of exo- and endo-cellulase using the SED1 anchoring region and its original promoter
Source: Biotechnol Biofuels. 2014 Jan 14;7:8. doi: 10.1186/1754-6834-7-8 (PMC3900695; doi:10.1186/1754-6834-7-8)

**Figure S1**

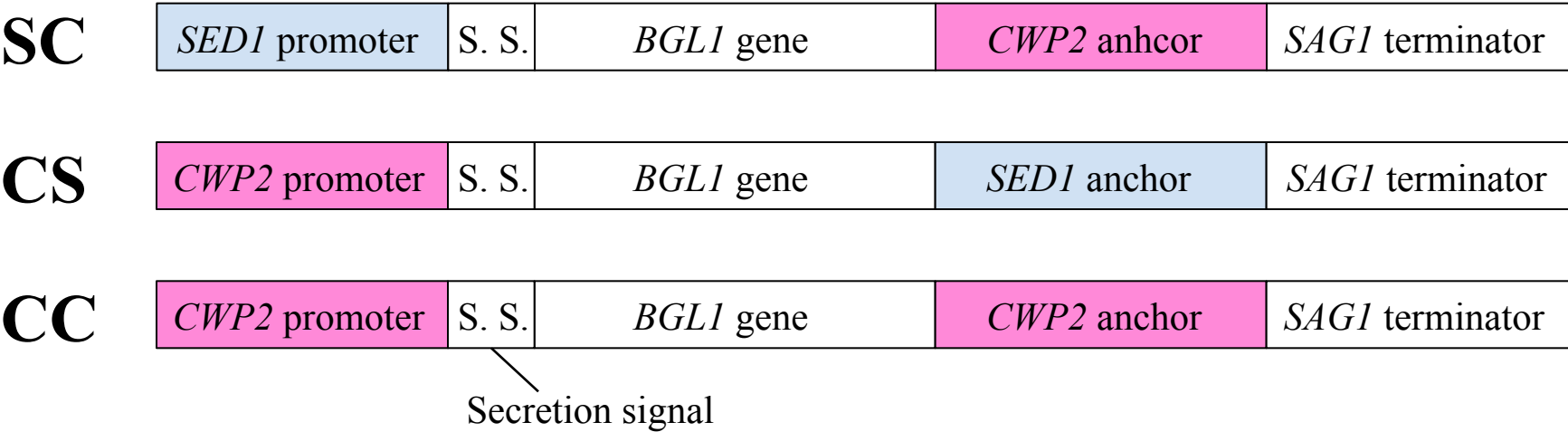

Supplement: Additional file 1: Figure S1 — Construction of novel gene cassettes with the S. cerevisiae CWP2 promoter and/or its anchoring region. The novel gene cassettes with the S. cerevisiae CWP2 promoter and/or its anchoring region were constructed based on the SS cassette for the yeast cell-surface display of β-glucosidase (BGL1). All cassettes have the secretion signal sequence of the R. oryzae glucoamylase gene and SAG1 terminator. [file 1754-6834-7-8-S1.pdf]

**Figure S2**

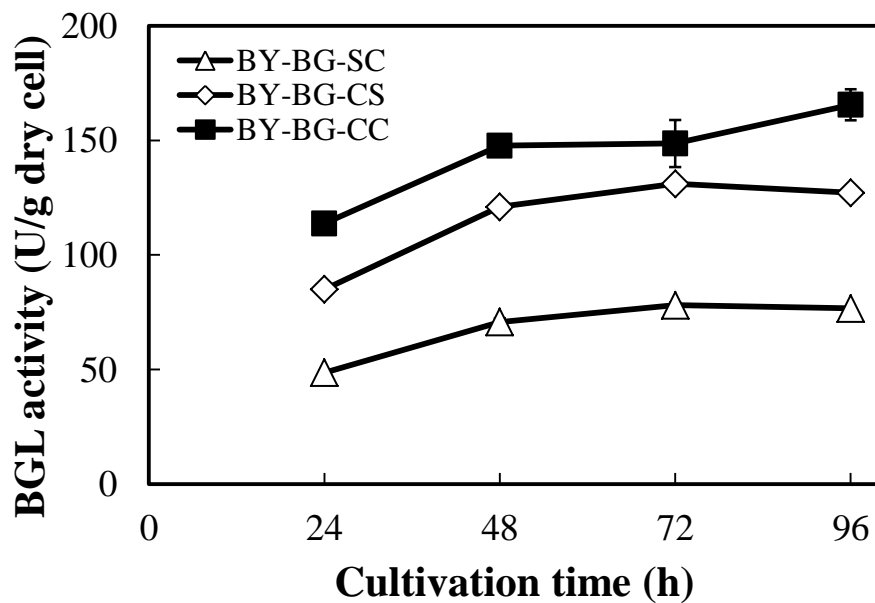

Supplement: Additional file 2: Figure S2 — Time course of β-glucosidase (BGL) activities of BGL-displaying strains (BY-BG-SC, CS and CC). Recombinant strains harboring gene cassettes shown in Additional file 1: Figure S1 were cultured under aerobic conditions at 30°C for 96 h. The culture broth was sampled every 24 h, and BGL activity in the cell was investigated as described in the Methods. Error bars indicate the standard deviations of three independent experiments. [file 1754-6834-7-8-S2.pdf]
